# A quantitative proteomic analysis of long-term memory

**DOI:** 10.1186/1756-6606-3-9

**Published:** 2010-03-23

**Authors:** David Rosenegger, Cynthia Wright, Ken Lukowiak

**Affiliations:** 1 Hotchkiss Brain Institute, Faculty of Medicine, University of Calgary, Calgary, Alberta, T2N 4N1, Canada; 2 Central Proteomics Facility, Department of Clinical Medicine, University of Oxford, Oxfordshire, OX3 7BN, UK

## Abstract

**Background:**

Memory is the ability to store, retain, and later retrieve learned information. Long-term memory (LTM) formation requires: DNA transcription, RNA translation, and the trafficking of newly synthesized proteins. Several components of these processes have already been identified. However, due to the complexity of the memory formation process, there likely remain many yet to be identified proteins involved in memory formation and persistence.

**Results:**

Here we use a quantitative proteomic method to identify novel memory-associated proteins in neural tissue taken from animals that were trained *in vivo *to form a long-term memory. We identified 8 proteins that were significantly up-regulated, and 13 that were significantly down-regulated in the LTM trained animals as compared to two different control groups. In addition we found 19 proteins unique to the trained animals, and 12 unique proteins found only in the control animals.

**Conclusions:**

These results both confirm the involvement of previously identified memory proteins such as: protein kinase C (PKC), adenylate cyclase (AC), and proteins in the mitogen-activated protein kinase (MAPK) pathway. In addition these results provide novel protein candidates (e.g. UHRF1 binding protein) on which to base future studies.

## Background

Learning and memory are separate but related processes each with its own 'rules and regulations'. Learning is the acquisition of a new behaviour, and memory the ability to store and recall this new information [1]. Memory formation is a complex process involving several necessary biomolecular steps. Long-term memory (LTM), which persists for days or even decades has been shown to require both transcription and translation (i.e. new protein expression) in order to occur [2-4]. In addition to transcription and translation LTM also typically requires the activation of second messengers by an appropriate signalling event, the amplification and translocation of this signal to the nucleus, and the transport of the newly synthesized proteins to the appropriate location in the cell [5,6]. It is apparent that memory formation and its maintenance is a process that requires the involvement of numerous proteins in order to occur, and the identification of the proteins involved is likely far from complete [7]. Owing to the complexity of memory systems, identifying those proteins that are causal to memory formation is no simple task.

In an attempt to work around the problem of the complexity of most memory systems (as based on sheer neuron numbers) we have chosen to use a reductionist model system of memory formation in *Lymnaea*. The fresh water snail *Lymnaea stagnalis *has been used in many studies of learning and memory. Owing to its relatively simple nervous system with large identifiable neurons, much progress has been made in characterizing the underlying neuronal circuitry that drives various behaviours [1,8,9]. One such relatively simple behaviour that has been studied in depth is aerial respiration. This breathing behaviour is driven by a 3-neuron central pattern generator (CPG), whose necessity and sufficiency have been shown [10,11]. This aerial respiratory behaviour can be operantly conditioned to form memories of varying duration, including long-term memories that require both the transcription and translation of new proteins to occur [12]. Additionally as this is a non-declarative form of memory, the site of physical storage of the memory should reside within the neurons that mediate the motor behaviour, i.e. the respiratory CPG. Right pedal dorsal 1 (RPeD1), a neuron in the respiratory CPG has been previously shown to be required for long-term memory, extinction, reconsolidation and forgetting [13,14]. This detailed knowledge of the neurobiology controlling respiration presents a unique opportunity to study LTM formation on the cellular level.

In this study we employ a quantitative proteomics technique to tissue taken from both control and animals trained to form a long-term memory. Using this protocol we wish to test whether it is possible to: 1) detect proteins previously associated with memory, and 2) detect any novel proteins not previously associated with the memory formation/storage process.

## Methods

### Animals

The fresh water snail *Lymnaea stagnalis *were used in the following experiments. Animals were bred and maintained at the facilities in the University of Calgary, from a colony initially set up at Vrije University in The Netherlands. Adult animals with a shell size larger than 20 mm were used in all experiments. Animals were maintained, and all experiments were performed at room temperature (~20-21°C).

### Training Protocols

Three sets of animals were used for this study, an operantly conditioned (LTM) group, a yoked control group, and a naïve group. *Lymnaea *are bi-modal breathers able to obtain their oxygen requirements cutaneously via diffusion across the skin, or aerially via the pneumostome (an opening into the lung). This allows for the aerial respiratory behaviour to be conditioned without compromising the animals survival [15]. This is done by placing individually labelled animals in water made hypoxic by bubbling N_2 _through for 20 minutes prior to training, in order to increase the aerial respiratory drive. Upon commencement of training the snails receive a gentle tactile stimulus to the pneumostome area each time aerial respiration is attempted. With training the animals learn to withhold this behaviour thus decreasing the number of attempted pneumostome openings. The LTM animals received three 45 min reinforcement sessions. Two 45 min training sessions on the first day separated by an hour, and a third 45 min training/memory test session on the second day. The yoked animals received the same hypoxia exposure and number of stimuli as the LTM group, except in a non-contingent manner (animals only receive stimulation if pneumostome is closed). Naïve animals received no treatment.

### Dissection

Animals were dissected one hour after the completion of the final training or control session. The animals were incapacitated by submersion in ice-cold saline, with the dissections also carried out in ice-cold saline. The central ganglia of the animals were quickly dissected out. Next the buccal ganglia were dissected away from the central ganglia followed by the removal of the left pedal (L.Pe.G), cerebral (L.Ce.G), and pleural ganglia (L.Pl.G) (Figure 2). This was performed in order to better isolate the brain regions containing the central pattern generator which controls aerial respiration [10], in an attempt to enhance the signal to noise ratio (cells where memory is stored to non-memory cells) of the samples. Upon completion of dissection the ganglia were placed in eppendorf tubes, and immediately snap frozen and stored at -80°C until use.

### Sample Preparation

0.5 mL of solubilization buffer (Urea-Thiourea-CHAPS buffer containing, 40 mM Tris, 5 M Urea, 2 M thiourea, 4% CHAPS, 10 mM DTT, 1 mM EDTA, 1 mM PMSF, and a Protein inhibitor cocktail) was added to each sample, and the samples were homogenised on ice using the loose pestle of a Dounce homogeniser. The samples were then mixed on a rotary mixer for 30 minutes and then centrifuged at 10 000 g for 10 minutes. The supernatant was transferred to a new tube and this was centrifuged for 45 minutes at 150 000 g to sediment any un-dissolved material. The supernatant was transferred to a new tube, and protein concentration was determined using the EZQ assay (Invitrogen). The samples were stored at -20 until used. The samples were then processed as follows for mass spectrometry analysis.

### Nano UPLC MS/MS

Protein samples were analyzed via an ultra-performance liquid chromatography tandem mass spectrometry (nano UPLC-MS/MS) protocol as previously described [16,17]. Briefly, the methanol-chloroform precipitated samples were subjected to in-solution tryptic digestion. Samples were then desalted using Sepak C_18 _columns (Waters, Milford, MA, USA). Next lyophilized peptides were resuspended (97% H2O, 3% acetonitrile, 0.1% formic acid) and subjected to the nano UPLC-MS/MS analysis using a Waters Nano-Acquity UPLC system with a 75-um inner diameter × 25 cm UPLC column and a 90 min gradient of 2-45% solvent B (solvent A: 99.9% H_2_O, 0.1% formic acid; solvent B: 99.9% acetonitrile, 0.1% formic acid; final flow rate 250 nl/min, 7000 psi), coupled to a Waters QTOF-premier tandem mass spectrometer (Waters, Milford, MA, USA). Data was acquired in high-definition MSE mode (low collision energy 4 eV, high collision energy ramping from 15 to 40 eV, switching every 1.5 seconds) and processed with ProteinLynx Global Server (PLGS version 2.2.5, Waters, Milford, MA, USA) to reconstruct MS/MS spectra by combining all masses with identical retention times. MS/MS spectra (peaklists) were searched against the Swiss Prot database using PLGS. Each sample was analysed in triplicate runs and were spiked with an alpha-enolase tryptic digest (125 fmol) as an internal standard. Quantitative changes in protein abundance, based on peptide ion peak intensities were analyzed using the Waters Expression Analysis Software (WEPS), which is part of the PLGS package.

### Western Blot and analysis

For the western blot analysis, the CNS of another set of animals were dissected out and snap frozen after treatment as described above (naïve n = 8, yoked n = 8, and trained n = 8). 0.5 mL of Nonidet-P40 (NP-40) buffer (containing, 20 mM Tris HCl pH 8, 137 mM NaCl, 10% glycerol, 1% NP-40, 2 mM EDTA, 1 mM PMSF, and a Pearce Halt protease inhibitor cocktail) was added to each sample, and the samples were homogenised on ice using the loose pestle of the glass homogeniser. The samples were then mixed on a rotary mixer for 30 minutes and then centrifuged at 10 000 g for 10 minutes. The supernatant was transferred to a new tube and this was centrifuged for 45 minutes at 150 000 g to sediment any insoluble material. Protein levels were standardised and samples were resolved using 10% SDS-PAGE gel. Proteins were transferred overnight at 4°C onto PVDF membranes. Membranes were blocked in 5% non-fat milk/TBST (20 mM Tris-HCl, 0.15 M NaCl, 01% TWEEN 20), and incubated with polyclonal anti-ADCY8 (1:5000) or anti-β-actin (1:10000) (Abcam, Cambridge, MA, USA). Membranes were then washed and incubated with an HRP-conjugated secondary antibody (1:10000). Western blots were visualized using ECL plus (GE healthcare, Piscataway, NJ, USA). Membranes were scanned using a Molecular Dynamics Storm 860 phosphoimager, and densiometric analysis was completed using ImageJ software.

## Results

### LTM training and control animals

**Figure 1:**
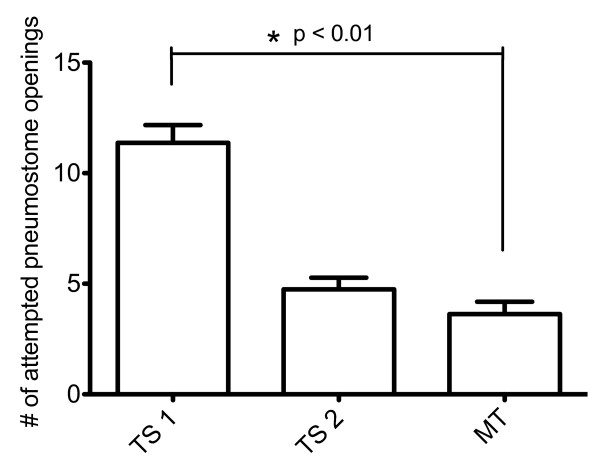
**LTM formation**. Animals given two 45 min training sessions separated by an hour (TS1 and TS2) have long-term memory when assayed 24 hours later (MT), as observed by a significant reduction in the number of attempted pneumostome openings (TS1 vs MT ANOVA F _(2,7) _= 45.24, p < 0.001).

Three groups of animals were used in this study; an operantly conditioned group (n = 8), a yoked control group (n = 8), and a naïve group (n = 8). The operantly conditioned animals received two 45-minute training sessions (TS1 and TS2) separated by an hour on day 1. We tested for memory 24 h later (MT). We define memory to be present if the number of attempted pneuomostome openings in the MT session is significantly less than that in TS1, and not significantly greater than that of TS2. As expected the operantly trained animals showed a significant reduction in the number of attempted pneumostome openings in the last session (MT) as compared to the first (TS1) (ANOVA F _(2,7) _= 45.24, p < 0.001), and thus we conclude that they successfully formed LTM (Figure 1). This data is consistent with previously published findings using this training regime [18]. Yoked control snails did not exhibit LTM (data not shown).

### Characterization of altered protein expression as a result of Long-term memory training

**Figure 2:**
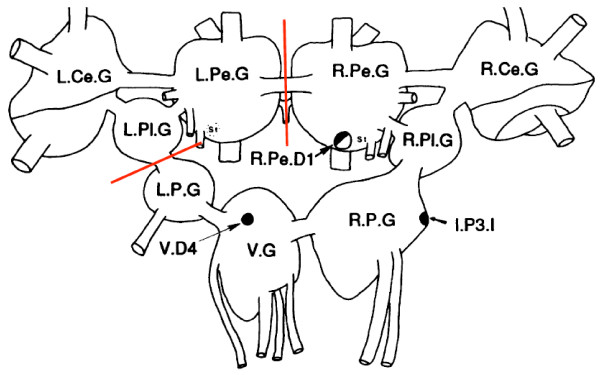
**Dissection of the Lymnaea CNS**. After excising the CNS from the animal, it was then further dissected by removing the left pedal, pleural and cerebral ganglia (L.Pe.G, L.Pl.G, and L.Ce.G respectively) via cutting at the indicated areas (red lines). The remaining ganglia of the CNS containing the neurons of the aerial respiratory CPG right pedal dorsal one (R.Pe.D1), input 3 interneuron (I.P3.I), and visceral dorsal 4 (V.D4) were processed for proteomic analysis. Figure adapted from Syed et al., 1990 [10].

**Figure 3:**
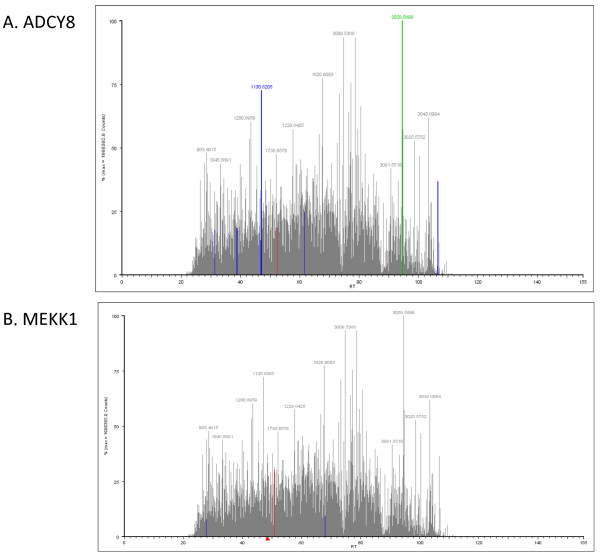
**Representative LC-MS/MS chromatograms**. LC-MS/MS chromatograms for ADCY8 (A) and MEKK1 (B).

Following behavioural testing for LTM, all snails (including the two control groups) had their nervous systems dissected out (Figure 2) and processed for proteomic screening as described in the methods. Briefly the snail brains were homogenized, proteins were extracted, subjected to a trypsin digestion and analyzed by inline liquid chromatography and mass spectrometry. Hundreds of proteins were positively identified in each sample (LTM = 503, Naïve = 506, and Yoked = 518). From these identified proteins there were found to be a large number that were significantly different between the LTM group and the controls (yoked and naïve). To better focus the presentation of the data we are reporting only those proteins that were found to be significantly different (increased/decreased, present/absent) in the LTM group as compared to **both **the naïve and yoked control groups. That is any protein found to be significantly different in the LTM group, showed a significant difference from both the naïve and the yoked groups for the identical protein, and in the same direction. Using these criteria there were 8 proteins that were found to be significantly more abundant in the LTM trained animal sample as compared to the controls, and 13 proteins which were significantly less abundant (Table 1). 19 proteins were found to be unique to the LTM trained animal sample, while 12 proteins were detected in both control samples but not in the LTM sample (Table 2). Included in the list of proteins that were up regulated in LTM animals were adenylate cyclase type 8 (ADCY8) and mitogen activated protein kinase kinase kinase 1 (MEKK1) whose chromatograms are shown in figure 3. These two proteins have previously been associated with the memory formation process. ADCY8 is membrane bound enzyme that catalyses the formation of cyclic AMP (cAMP) in response to stimulus evoked entry of calcium into neurons. ADCY8 knockout mice show deficits for memory retention in object recognition and passive avoidance tasks [19], as well as deficits in the formation of mossy fiber long-term potentiation (LTP) in the hippocampus [20]. MEKK1 is a serine/threonine kinase, and a member of the MAPK signal transduction pathway. MEKK1 has been shown to be an activator of both the extracellular signal-regulated kinases 1 and 2 (ERK1/2) and the c-Jun N-terminal kinase 2 (JNK 2) pathways [21], with ERK activity being necessary for the induction of LTP [22], and the formation of various kinds of memories [23-25].

**Table 1:** Proteins significantly up/down regulated in LTM vs. controls

Protein description	Accession number	PLGS Score	Mass kDa.	No. of MS/MS Spectra	Seq. Coverage %	Fold change LTM vs. Naive	p-value	Fold change LTM vs. Yoked	p-value
Kinesin like protein KIF14	Q15058	109.68	186.49	21	13	1.12	< 0.01	1.19	< 0.01
Canalicular multispecific organic anion transporter	O15438	74.4	169.34	24	23	1.11	0.01	1.18	< 0.01
UHRF1 binding protein	Q6BDS2	65.7	159.49	10	8	1.16	< 0.01	1.25	< 0.01
Putative hexokinase HKDC1	Q2TB90	53.72	102.52	2	2	1.19	0.01	1.39	< 0.01
Uncharacterized protein C2orf54	Q08AI8	49.33	49.57	11	31	1.12	0.05	1.16	0.01
Adenylate cyclase type 8	P40145	87.68	140.12	35	36	1.22	< 0.01	1.11	0.03
Bile salt export pump	O95342	43.39	146.41	1	1	1.12	0.04	1.11	0.03
Mitogen activated protein kinase kinase kinase 1	Q13233	59.93	164.47	13	12	1.11	0.01	1.11	0.04
5 azacytidine induced protein 1	Q9UPN4	91.48	122.06	18	17	0.93	< 0.01	0.93	< 0.01
Zinc finger protein 28 homolog	Q8NHY6	79.58	98.71	13	14	0.93	0.03	0.8	< 0.01
Kelch repeat and BTB domain containing protein 5	Q2TBA0	37.8	69.26	9	16	0.85	0.01	0.78	< 0.01
Diacylglycerol kinase kappa	Q5KSL6	36.22	141.83	14	15	0.88	< 0.01	0.88	< 0.01
Phosphatidylinositol 4 phosphate 3 kinase C2 domain containing alpha polypeptide	O00443	90.69	190.68	24	17	0.92	0.04	0.88	0.01
Acyl CoA dehydrogenase family member 10	Q6JQN1	67.81	118.83	11	11	0.93	0.03	0.92	0.01
Arf-GAP with SH3 domain, ANK repeat and PH domain-containing protein 1	Q9ULH1	60.8	125.47	15	15	0.88	0.03	0.88	0.01
ATP binding cassette sub family A member 3	Q99758	47.19	191.36	16	13	0.88	0.01	0.92	0.01
Cytochrome P450 26A1	O43174	41.39	56.2	6	14	0.87	0.03	0.89	0.02
Tyrosine protein kinase JAK2	O60674	56.35	130.67	10	9	0.92	0.03	0.9	0.03
NTPase KAP family P loop domain containing protein 1	Q17RQ9	52.69	67.78	13	26	0.83	0.01	0.88	0.03
Ectonucleotide pyrophosphatase phosphodiesterase family member 2	Q13822	47.65	99	9	14	0.83	0.05	0.83	0.03
Multidrug resistance associated protein 9	Q96J65	59.07	152.3	1	1	0.86	0.02	0.94	0.05

Accession number for Swiss Prot.

PLGS Score is calculated by the Protein Lynx Global Server (PLGS 2.2.5) software using a Monte Carlo algorithm to analyse all available mass spec. data and is a statistical measure of accuracy of assignation. A higher score implies greater confidence of protein identity [16].

**Table 2:** Proteins unique to LTM and control conditions

Protein description	Mass kDa	Accession number	PLGS Score	No. of MS/MS spectra	Seq. coverage %	Unique to (LTM or yoked/naïve)
Protein kinase C epsilon	83.67	Q02156	53.24	10	16	LTM
Centrosomal protein of 63 kDa	81.34	Q96MT8	47.33	11	18	LTM
Ankyrin repeat and IBR domain containing protein 1	122	Q9P2G1	40.79	8	9	LTM
Bcl 2 related proline rich protein	36.82	Q9HB09	40.7	8	21	LTM
Olfactomedin like protein 1	45.92	Q6UWY5	40.69	4	9	LTM
Zinc finger protein 271	75.54	Q14591	40.68	6	10	LTM
Importin 9	115.96	Q96P70	40.27	14	20	LTM
Uncharacterized aarF domain containing protein kinase 5	65.9	Q3MIX3	39.92	11	24	LTM
Gamma parvin	37.49	Q9HBI0	38.99	6	20	LTM
Nucleolar protein 11	81.12	Q9H8H0	38.88	10	18	LTM
39S ribosomal protein L17 mitochondrial	20.05	Q9NRX2	38.59	8	42	LTM
Chromobox protein homolog 6	43.9	O95503	37.55	7	18	LTM
Zinc finger protein 101	50.34	Q8IZC7	37.42	3	7	LTM
Transmembrane protein 68	37.43	Q96MH6	36.96	8	28	LTM
Ras association domain containing protein 5	47.09	Q8WWW0	36.83	6	20	LTM
Peroxiredoxin 4	30.54	Q13162	36.11	6	26	LTM
Solute carrier family 25 member 35	32.44	Q3KQZ1	35.33	7	29	LTM
Golgin subfamily A member 5	82.99	Q8TBA6	35.2	9	15	LTM
Putative uncharacterized protein CXorf55	114.94	Q8N7X1	35	7	8	LTM
Ras GTPase activating protein 1	116.4	P20936	60.25	17	18	N/Y
Elongator complex protein 1	150.25	O95163	52.49	11	12	N/Y
Alpha type platelet derived growth factor receptor precursor	122.67	P16234	49.83	12	10	N/Y
Evolutionarily conserved signaling intermediate in Toll pathway	49.15	Q9BQ95	48.69	3	8	N/Y
Vacuolar proton translocating ATPase 116 kDa subunit a isoform 4	96.36	Q9HBG4	48.44	10	16	N/Y
Exportin T	109.96	O43592	45.66	11	16	N/Y
C type lectin domain family 4 member F	65.52	Q8N1N0	44.32	8	15	N/Y
Transmembrane protein 16B	113.97	Q9NQ90	43.27	8	9	N/Y
Hepatocellular carcinoma down regulated mitochondrial carrier protein	33.44	Q6Q0C1	41.69	7	20	N/Y
Cell division cycle 7 related protein kinase	63.89	O00311	41.36	6	13	N/Y
Myotubularin	69.93	Q13496	39.76	7	13	N/Y
Aladin Adracalin	59.57	Q9NRG9	39.32	6	15	N/Y

Accession number for Swiss Prot.

### Western blot

**Figure 4:**
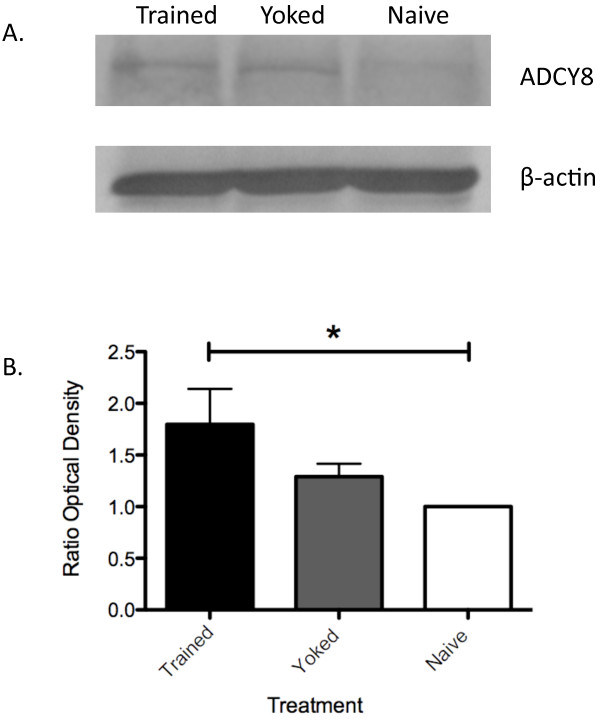
**ADCY8 is significantly up-regulated in trained animals**. A) Animals trained to form a LTM have increased levels of ADCY8 as compared to Naïve animals by western blot analysis B) Average change in ADCY8 expression (n = 4, LTM vs. p < 0.05).

To validate the results of the quantitative MS experiments, we performed a set of western-blot experiments on one of the proteins found to be up-regulated in the LTM sample; ADCY8. For this experiment three new sets of samples were prepared, as previously described, and four separate gels and blots were done to allow for quantification. As shown in figure 4, animals trained to form a LTM showed a significant increase in the amount of ADCY8 as compared to naïve animals. While the western blot results show a similar pattern of results when compared to the UPLC-MS/MS data, they differed slightly as the change in ADCY8 expression between yoked and trained animals did not reach statistical significance by western blot analysis. This difference may be attributed to the much higher precision and sensitivity of measurement made by the UPLC MS/MS method.

## Discussion

In this study we used quantitative proteomic methods to identify changes in protein expression in *Lymnaea stagnalis *trained to form a long-term memory as compared to controls. At the time point measured (1 hr after the final training session), we observed that the abundance in 21 proteins was significantly altered by the training procedure when compared against both control groups. In addition to changes found in those 21 proteins, we also identified 19 proteins that were found to be uniquely detected in the LTM group and not in either control, and conversely we found 12 proteins that were expressed in both the control groups but not found in the LTM group.

Several of the proteins identified have already been implicated in various learning/memory paradigms. For example in the present experiment adenylate cyclase type 8 (AC8) was found to have increased expression in LTM animals as compared to the controls. AC8 is stimulated by Ca^2+^/calmodulin and acts to increase the production of cyclic adenosine monophosphate (cAMP), which in turn activates protein kinase A (PKA) that can phosphorylate and bind to promotor regions of DNA. Previously it has been shown that AC1/AC8 double knock out mice display hippocampal dependent LTM deficits [26], and a more recent study has shown AC8 to be required for working/episodic memory as well as the acquisition of newer spatial information in mice [19].

We also found that protein kinase C-epsilon (PKC-e) was uniquely expressed in LTM trained animals but not detected in either of the controls. PKC enzymes operate by phosphorylating various target proteins and therefore can modulate their actions. PKC epsilon has been shown to modulate Na^+ ^channel activity and neurotransmitter release in hippocapal neurons [27]. In numerous studies the PKC family of enzymes have been shown to be necessary in various forms of synaptic plasticity, and memory formation, with the epsilon isoform required for long-term potentiation (LTP) in the mossy fiber CA3 hippocampal pathway [28]. Additionally in Lymnaea, the PKC agonist bryostatin will allow a LTM to form with a reduced training paradigm, and increase the duration of the memory formed [29].

In addition to the proteins for which a role in the memory process has already been established, we identified some whose roles are less than clear. One example is the ubiquitin like containing PHD and RING finger domains 1 binding protein (UHRF1 binding protein). UHRF1 is a protein that is involved in the maintenance and replication of global and local DNA methylation in vivo [30]. DNA methylation has been shown to be an important step in memory formation [31], and thus UHRF1 may be one of the proteins involved in mediating this alteration in the histone code that takes place when a memory is formed.

The results of our experiments would indicate that the formation of a long-term memory is a very complex process involving a number of diverse cellular systems. Most research on the subject has historically focused on the various mechanisms of cell signalling, transcriptional regulation, and protein synthesis. Our proteomic data would suggest that memory formation involves changes in a number of cellular systems not limited to; energy/cellular metabolism and ion gradient regulation, cytoskeletal organization, secretion, cell adhesion, and chromatin modification. In future experiments we hope to use this data to design experiments allowing us to test the necessity of these various systems/proteins for the memory formation process. As a brief example the chromatin modifying chromobox protein homolog 6 found to be present in only LTM trained animals, could be inhibited by RNAi prior to training to determine if memory can still be produced.

## Conclusions

In this study we were able to use a quantitative proteomic approach to identify changes that occur in protein expression after an in vivo training paradigm that results in long-term memory formation.

## Competing interests

The authors declare that they have no competing interests.

## Authors' contributions

DR designed the study and carried out the behavioural experiments, tissue isolation, western blot experiments, data analysis, and manuscript preparation. CW performed the proteomic analysis and participated in the data interpretation and analysis, and aided in the revision of the manuscript. KL participated in the design and coordination of the project, and manuscript revision. All authors read and approved the final manuscript.
